# Ancient Medicine

**Published:** 1860-11

**Authors:** 


### Editors' Table.

Ancient Medicine. — Of the two great divisions of medical inquirers, one is continually engaged in opening out new paths and making observations and discoveries; the other is retrospective in its labors, and gathers up and arranges the materials of olden time, which had been neglected or forgotten, and shows often their resemblance to, and harmony if not identity with, the more recent delvings of modern science. We should be loth to be compelled to ally ourselves to one of these divisions in such a way as to imply abandonment of the other; although many, we are afraid they constitute the majority of the profession, unite themselves ostensibly with the first or progressive party, and do not think it worth while even to look over their shoulders at the road that has been traveled by their predecessors. Neglect of this kind is not without its penalty. A clear knowledge of the country that has been gone over gives greater confidence in making explorations in advance, and prevents the recurrence of many vexatious mistakes—a wandering in by-paths, the like of which had been shown by former experience to be misleading and unproductive.

It must be confessed, however, that retrospective lore is too frequently brought before us in its driest and most forbidding aspects. A tangled thread of speculative argument runs through the whole period of medical history, and it is not unraveled by polyglot criticism, straining and pulling in different directions. Men preferred to wander through a dark labyrinth, in the vague hope of finding concealed treasure, in place of gathering fruits and succulent roots, the products of the upper earth, but scattered abroad without rule or method. Ancient medicine abounds in suggestions full of applicable philosophy and plain precepts, enforced by pertinent and striking facts, easily separable from the superstitions, fictions, and theories with which they are often associated. But even the superstitions and fictions which belong to the first or mythical period of ancient medicine, evinced, on the part of those who gave them shape and currency, a large comprehension of the aids and alliances of the healing art. Apollo, the god of music, he of the tuneful lyre and vengeful bow, was also the god of medicine. Chiron the Centaur, the son of Saturn, and the first surgeon if not the first physician, was the preceptor of Æsculapius, Hercules, Theseus, Jason, and other heroes of early Greek traditions. He taught music as well as medicine, and astronomy in addition to philosophy. Æsculapius, among his curative means, had recourse to songs and melody, and amusing exhibitions, for the purpose of allaying the excitement and disorders produced by indulgence in strong passions. Hercules, the mere type of brute force in the conceptions of most readers, was meant to show also the union of mind with muscle, as in two of his twelve labors, viz., destroying the many-headed hydra of Lerna, and in cleansing the Augean stables. In both of these feats he showed himself to be the pupil of Chiron, and the practiced sanitarian: in the first case by draining the marshes and exposing them to the sun, and thus removing the causes of disease, and destroying the so-called hydra; in the second, by turning the River Peneus or Alpheus through the stable so as to wash away its accumulated filth. Poetical fictions these, but withal pleasant and instructive.

Hygeia, the daughter of Æsculapius, and goddess of health, was not worshiped in crowded cities. Her temples were amid groves and streams, and they crowned heights from which the eye could survey the varied landscape around. The inmates and visitors to the temples of Æsculapius or temples of Health, Asclepias, thus favorably situated, enjoyed all the advantages of pure air and the superadded appliances of baths, frictions, gymnastic exercises, and riding on horseback. In the instance of the temple of Epidaurus, their senses were agreeably impressed and their minds diverted by music, recitations, and dramatic performances in a theatre in the vicinity. We may smile at the mummeries and jugglery practiced on their patients, especially in the quiet and silence of the night, in the chambers of the temple, on the pretence of placing them under divine influence. But we cannot deny that the sights and sounds created at these times, following also imposing religious ceremonies during the day, could not fail to affect powerfully the imagination, and, by exciting faith and hope, produce a strong effect on all the functional disorders of the nervous system. Could the science of the present day, calling in religion to its aid, suggest a better preparation for subsequent treatment than ablutions, abstinence, and prayers enjoined on the sick previously to their being received into the temple? Does any sanitarium, fashionable watering-place, or hydropathic establishment present such a bill of fare for the choice and use of the invalid—such a union of applied physiology and psychology—which shall take cognizance of man's mixed corporeal and mental nature?

The doctrines of Hippocrates on the elements and humors, and on certain crises explained and commented on by Galen, were for many centuries reverently received in the schools in apparent forgetfulness of his admirable treatises on Air, Water, and Places, and on Diet. His Aphorisms also were long regarded as the capstone of the scientific edifice of medicine, the most sublime effort of medical genius. It was exacted by the faculty of Paris until a short period, as an indispensable condition for the success of the candidates for the doctorate, that they should insert in their theses a certain number of these Hippocratic aphorisms. The far more valuable treatises by the Father of Medicine on Prognostics and on Epidemics, containing direct and available lessons, failed to attract any such deferential regard. The matters described in these treatises and in the ones first named should induce us to look back on ancient medicine as available for much positive instruction, divested of the mystic, the obscure, and the erroneous, which, in the minds of many, alone characterize it.

The abstruse speculations of Galen, his doctrine of the four humors, and of eight leading constitutions, his explanations of the power of medicines depending on their general qualities of heat and cold, moisture and dryness, were taught and "implicitly followed by all the physicians of Greece who came after Galen, and indeed by all the physicians of Asia, Africa, and Europe, for at least fifteen hundred years after his time."

His *Ars Medica*, as we had occasion to relate in the first volume of this Journal, was, for centuries, the text-book on which the students of Salernum and of other schools of the middle ages were examined, before receiving permission to practice. If the veneration for antiquity has retarded the progress of medicine in times preceding our own, it does not follow that an entire neglect of it is to be encouraged now. We may deprecate the erroneous philosophy and faulty discrimination which led men's minds to dwell on obscure hypotheses and wordy speculations, to the exclusion of that which was clear, plain, and practical, while we, with better lights and more discernment, can make better selections from the very same stores of antiquity. We do not turn away from Milton and refuse to read, and study too, his "Paradise Lost," because his "Paradise Regained" was a poem of inferior merit. Of the five hundred treatises by Galen, some of them, it is true, not larger than small pamphlets, the greater number of those which are preserved may be cast to one side; but there will still remain for our edification his admirable books on Semeiology and Hygiene. In the latter, especially, he was not only more definite, full, and perspicuous, than any writer who preceded him, but in some points remains superior to any of his successors. The prominence which he gave to Hygiene, as displayed in his treatises on the Preservation of Health, and *De Sanitate Tuendâ*, and on the "Properties of Aliments," is consistent with his definition of medicine, viz.: the preservation of health and the cure of disease. The first part of this definition was soon overlooked by Galen's successors; and it is only just now beginning to arrest the attention of medical men, who, for the most part, think that their duty is discharged when they pay the last visit and have written the last prescription for their convalescent patients.

The great teacher of Pergamos has the merit of originality in treating the following four subjects, viz: 1. On infancy; 2. On old age; 3. On temperaments; 4. On those who are not masters of their time. His inculcations on these themes have still the freshness of novelty if we may judge from the general neglect with which they continue to be viewed. Infants, he says, should be fed with their mothers' milk in preference to that of a stranger. This ought to be their sole nourishment until they get their first teeth. Attention ought to be paid to whatever causes them to cry or gives rise to much restlessness. Every morning, and on an empty stomach, they should be bathed in tepid water, and afterwards well dried and rubbed. He reprobates the practice of the people of the North, who plunge their children, at the moment of their birth, into cold water. Making one of those flings of which Galen was too fond, he says he has no desire to write for those Germans and those Barbarians any more than for bears and lions. He lays much stress on the great care that ought to be taken of nurses, in regard to their regimen, exercise, sleep, etc., if it be desired that they should give good milk. Nothing, he assures us, is more necessary to young children than that they should breathe a pure air, and hence they ought not to be kept in close rooms. How little consonant with this advice is the practice of many mothers, even in the present physiological age of Liebig, Dumas, and others, in allowing their infants to sleep in their arms a good part of the night, and so covered up as to allow of the least possible access of air—barely enough to prevent suffocation, while the bedroom itself is nearly air-tight, at any rate destitute of all appropriate means of ventilation. Galen's directions extend to the removal of children from marshy places, and the exhalations from great cities.

Among the hygienic precepts to old persons, we may select the following: To excite the functions of the skin by frictions and the flesh-brush—bathing was, in that age, a matter of course; to take, without fail, moderate exercise, enough to keep up the strength; but to avoid carefully the carrying it to the extent of producing fatigue, which exhausts and emaciates; to use fluid and invigorating food, and to drink generous wine, which shall be at the same time diuretic; and be very attentive to keeping the bowels open. The wine of that period, it will be remembered, was really the product of fermentation of the juice of the grape, without the foreign addition of alcohol, which the genius of evil had not yet shown man how to procure by distillation. Galen attached great importance to a knowledge of the temperaments; deeming it to be necessary for a proper adaptation of hygienic agents, and, more particularly, of the modifications of regimen to individual cases. He enlarges on this topic, on which we cannot follow him without being led into details and theoretical disquisitions for which we have no room. We may say, however, that Galen's divisions of the temperaments have been, in the main, retained even to the present time.

As relates to persons in public life, men of letters and others whose accumulated engagements prevent their disposing of themselves and of their time as they might wish, Galen lays down for their guidance the three following rules: First, whenever they are tasked to any unusual effort, in the way of mental labor and study, they must be more than usually temperate; secondly, they are to use the simplest diet, and that which is most easy of digestion; and, finally, they must give some time, however short, to daily exercise, or, if that is impossible, to lose a little blood to prevent plethora, and also to take from time to time a mild purgative, to cleanse the stomach and bowels from impurities which accumulate in these passages. Failing to attend to these rules, persons thus situated can hardly fail to escape serious attacks of disease.

In commenting on his hygienic directions for those whose minds are kept on the stretch in active intellectual pursuits, and, it might be added, in commercial and other business walks, we must remember that Galen was no mere literary recluse or ascetic, intent on mortifying the appetites from religious considerations, or in accordance with the self-denials of stoical philosophy. He was, on the contrary, a man of the world, a great traveler, the physician of four Roman emperors, and, withal, himself an ardent and industrious student, who knew from personal experience the conditions most favorable for success in intellectual exercises. Up to the twenty-eighth year of his life, he scarcely passed one of them without being afflicted with some disorder; but, having studied his own constitution and fixed on the methods of improving it, he reached, after a time, an excellent state of health and old age—the effects of preserving a strict regimen both in diet and exercise. He practiced in his own person a rule, which he was the first to introduce as a part of hygiene, viz., the government of the passions.

Hippocrates and Galen are so commonly spoken of together, and the latter is so noted as the most extensive commentator and warmest eulogist of the Coan sage, that we are apt to forget the long period which separates these eminent Greek authors, and the fact of the Latin Celsus having lived in earlier times than Galen. Celsus was called the Latin Hippocrates, of whose opinions and practice he presents the best summary extant, and on this account alone, apart from his own merits as an eclectic writer, he would furnish ample inducements for our looking back, with respect, on ancient medicine. His counsels for the preservation of the health of those who already enjoy this blessing, and for the course to be pursued by the dyspeptic, in the first and second chapters of the first of the eight books of his celebrated work *de Re Medica*, are worthy of careful and continued perusal; they contain, in brief, nearly all that is now-a days expanded into volumes. The remarks of Celsus on the pulse are discriminating and practical. In them, repeating the language of a pleasant and learned historian of medicine, "there is much to commend, much to remember, and much to adopt." They are in strong contrast with the long, elaborate, and somewhat fanciful disquisitions on the pulse in Galen's several tracts, seventeen in number, on Sphygmology.

